# Mercy Hospital

**Published:** 1879-03

**Authors:** F. E. Waxham


### ARTICLE VIII.

### MERCY HOSPITAL.

(Reported by F. E. WAXHAM, M.D.)

Case I.—*Retro-Œsophageal Abscess.*

Mary B., æt. 27, entered the hospital June 11th, 1878. She complained of a very severe cough, which seemed to be paroxysmal in character, and had existed only for a few days. These paroxysms were very distressing, and seemed to be aggravated by exercise. Anti-spasmodics were given, but without affording relief. In a few days there began to be obstruction to the passage of food into the stomach. Pain became excessive. The patient said she felt as if there was a large swelling in the œsophagus. In a short time she began to vomit, and everything taken into the stomach was rejected. She refused to take enemas, and became greatly exhausted. Her pulse fell to 44 per minute, and it was thought that she could hardly live from one day to another. It was supposed that she was suffering from cancer at the cardiac extremity of the stomach, and she was placed upon appropriate treatment.

July 11th. Vomited about a pint of sanguineo-purulent matter. She also had several purulent discharges from the bowels. July 12th. Very much better; vomiting had entirely ceased. Her appetite returned, and food retained. July 20th. Discharged cured.

Case II.—*Laryngitis with Œdema of Glottis.*

Frederick Hager, æt. 43, entered the hospital July 8th, suffering from simple laryngitis with œdema. Breathing extremely difficult and irregular. He was obliged at times to stand, place his hands to his sides, and struggle for breath. Voice hoarse and jerky; some cough. The inspiration required much more effort than the expiration. The disease commenced four days before entering, but was quite mild until the day previous, when he suffered greatly from dispnœa. His attending physician stated that he received during the day 0.48 grams of croton oil without effect. He also received a topical application of a strong solution of nitrate of silver, which increased the difficulty of respiration, threatening suffocation.

Upon entering the hospital a mustard plaster was applied to the chest, and a solution of persulphate of iron, 4 grams to water 64 grams, applied to the larynx and glottis by means of a sponge attached to a staff. This greatly increased the dispnœa for the time, but was frequently repeated, and seemed to afford relief. All preparations were made for performing tracheotomy, and the patient closely watched during the night.

July 9th. Considerably better; still continued the application of the persulphate of iron every two or three hours. July 12th. Considered out of danger. July 13th. Greatly improved. July 15th. Left the hospital cured.

Case III.—*Psoriasis Diffusa.*

Dennis Donahue, æt. 50, laborer. The patient entered the hospital October 9th, with general inflammation of the skin, with profuse desquamation, the cuticle coming off in the form of pearly white scales from head to foot, and leaving the cutis very red. Bowels generally regular; appetite good. General health fair. Temperature normal. Pulse 108. Last spring the patient was attacked with lichen tropicus, extending over the entire body. Following this was an attack of chills and fever. The entire skin became dark in color, and the epidermis came off in large patches. The skin then became almost natural, scaling only on portions of the body. About a month previous to entering the hospital, the disease rapidly spread over the entire body, and since then complete desquamation has been taking place. Patient complained of a constant itching, and was continually rubbing off the scales. These were rapidly reproduced; one pint of these scales were collected during a period of twelve hours.

*Treatment*—He was directed to take Fowler's solution, 0.48, after meals, and to anoint his body thoroughly at night with oleum ricini. Oct. 12th. As he did not sleep well, he was directed to take 0.6 chloral hydrate, with 1.2 potassic bromide at night.

October 25:

| | | |
|---|---|---|
| ℞ | Olei Morrhuæ | 200 |
| | Hypophos. sodæ | 10 |
| | Hypophos. calcis | 10 |
| | Aquæ calcis | 200 |
| | Gummi acaciæ. q. s | |

M. et sig. Tablespoonful after meals.

For local application, he received a lotion of acetate of lead one gram, and borax two grams, in 100 grams of water.

Nov. 2d. Face begins to look quite natural, also his breast and arms, but his hands and feet show but little improvement.

Nov. 4th. The patient left the hospital, much improved.

